# Year-Round Respiratory Syncytial Virus Transmission in The Netherlands Following the COVID-19 Pandemic: A Prospective Nationwide Observational and Modeling Study

**DOI:** 10.1093/infdis/jiad282

**Published:** 2023-07-21

**Authors:** Yvette N Löwensteyn, Zhe Zheng, Neele Rave, Michiel A G E Bannier, Marie-Noëlle Billard, Jean-Sebastien Casalegno, Virginia E Pitzer, Joanne G Wildenbeest, Daniel M Weinberger, Louis Bont, for the Surveillance of Pediatric REspiratory Admissions in Dutch hospitals (SPREAD) Study Group, Marlies Vermaas-van Putten, Elly Smit-Kleinlugtenbeld, Marieke Peetsold, Martijn van der Kuip, Hans van Goudoever, Britt van Keulen, Anouk Boot, Robin Kloos, Sandy van Gool, Yvonne Snepvangers, Anke Kuijpers, Negassi Menelik, Stephanie de Crom, Carien Miedema, Gavin ten Tusscher, Jet van Giessen, Ronald de Moor, Marianne Faber, Mijke Breukels, Vincent Jaddoe, Liesbeth Duijts, Claire Lutterman, Ilka Vink, Gerdien Tramper-Stranders, Annemarie Oudshoorn, Astrid Ritman, Gerdien Dubbink-Verheij, Jantien Bolt, Cagri Cakir, Edwin Rietveld, Jolita Bekhof, Edmond Rings, Jara de Swart, Gertjan Driessen, Rienus Doedens, Lieke Nijssen, Lonneke van Onzenoort-Bokken, Ruud Meijneke, Machteld van Scherpenzeel, Tina Faber, Femke de Groof, Sarah Schouten, Julia van de Zande, Monique op de Coul, Stefanie Henriet, Kim Stol, Maaike van Rossem, Monique Jacobs, Marlies van Houten, Roy Zuurbier, Frans Plötz, Andra de Vries, Rinske van der Heide, Anneke van Boekholt, Gieneke Gonera de Jong, Amara Nassar-Sheikh Rashid, Manouck Roelofs, Károly Illy, Naomi Reijmerink, Stefan van Dorth, Saskia Schipper, Philippe Rosias, Anne Teirlinck

**Affiliations:** Department of Pediatric Immunology and Infectious Diseases, Wilhelmina Children's Hospital, Utrecht, The Netherlands; Public Health Modeling Unit, Department of Epidemiology of Microbial Diseases, Yale School of Public Health, New Haven, Connecticut; Department of Pediatric Immunology and Infectious Diseases, Wilhelmina Children's Hospital, Utrecht, The Netherlands; Department of Pediatric Respiratory Medicine, Maastricht University Medical Center, Maastricht, The Netherlands; Department of Pediatric Immunology and Infectious Diseases, Wilhelmina Children's Hospital, Utrecht, The Netherlands; Department of Microbiology, Hospices Civils de Lyon, Lyon University Medical Center, Lyon, France; Public Health Modeling Unit, Department of Epidemiology of Microbial Diseases, Yale School of Public Health, New Haven, Connecticut; Department of Pediatric Immunology and Infectious Diseases, Wilhelmina Children's Hospital, Utrecht, The Netherlands; Public Health Modeling Unit, Department of Epidemiology of Microbial Diseases, Yale School of Public Health, New Haven, Connecticut; Department of Pediatric Immunology and Infectious Diseases, Wilhelmina Children's Hospital, Utrecht, The Netherlands

**Keywords:** respiratory syncytial virus, COVID-19, epidemic timing, seasonality, waning immunity

## Abstract

We initiated a nationwide prospective study to monitor respiratory syncytial virus (RSV)–related pediatric hospitalizations in 46 hospitals throughout the Netherlands between May 2021 and August 2022. We showed year-round RSV transmission in the Netherlands after an initial 2021 summer outbreak. The pattern was unprecedented and distinct from neighboring countries. We extended a dynamic simulation model to evaluate the impact of waning immunity on pediatric RSV hospitalizations in the Netherlands using 4 different scenarios. Our results suggest that the observed continuous RSV transmission pattern could be associated with waning immunity due to the period of very low RSV circulation during the COVID-19 pandemic.

## Teaser

In a nationwide prospective multicenter study, we show year-round respiratory syncytial virus transmission after an out-of-season outbreak in the Netherlands following the COVID-19 pandemic. Our extended dynamic simulation model suggests that this novel continuous transmission pattern could be associated with waning immunity.

Before the coronavirus disease 2019 (COVID-19) pandemic, respiratory syncytial virus (RSV) epidemics occurred annually during winter in temperate climates (Supplementary Figure 1). During winter 2020–2021, RSV was virtually absent following the implementation of COVID-19–related nonpharmaceutical interventions (NPIs) [1, 2]. After NPIs were relaxed, various patterns of reemergent RSV epidemics were observed in different countries. Several studies suggested that school reopening is associated with increased RSV activity [1, 3]. However, little attention has been paid to the impact of increased RSV susceptibility in children and adults due to low RSV exposure during the pandemic (“immunity debt”) on the patterns of resurgence [4].

Using a combination of prospective surveillance data from the Netherlands and simulation models, this study aimed to (1) describe the unusual endemic pattern of RSV between May 2021 and August 2022 and (2) illustrate the impact of waning population immunity on the timing, intensity, and persistence of the reemergent RSV epidemic. This study provides an opportunity to advance our understanding of the drivers of reemergent RSV epidemics.

## METHODS

### Study Design

We initiated a prospective nationwide surveillance study (Surveillance of Pediatric REspiratory Admissions in Dutch hospitals [SPREAD]) in which real-time data are collected on RSV-related pediatric hospitalizations in 46 hospitals throughout the Netherlands (Supplementary Table 1). In 10 hospitals with standard-of-care RSV testing, clinical patient data are collected (Supplementary Figure 2). For this article, we collected data from 3 May 2021 (week 18) until 4 September 2022 (week 35). Age-stratified data were collected from October 2018 until August 2022. The study population included all children aged <2 years who were hospitalized with RSV bronchiolitis in participating hospitals.

Collaborators of participating hospitals were requested to share data on a weekly basis. Follow-up and data verification were performed by the study team to ensure data quality. Data were entered into the Castor Electronic Data Capture system [5]. We analyzed potential differences in the age distribution of patients during the following periods: pre–COVID-19 (2018–2019 and 2019–2020 winter seasons [October–April]), COVID-19 summer outbreak (May–August 2021), and COVID-19 endemic phase (September 2021–August 2022). Retrospective pre–COVID-19 data were collected using diagnosis treatment combination (DBC) codes (3210: RSV bronchiolitis; 3208: lower respiratory tract infection; 3104: upper respiratory tract infection). RSV-positive admissions were manually confirmed using patient files. We used the Mann–Whitney *U* test to determine statistically significant differences (defined as *P* < .05) between subgroups. Analyses were performed with SPSS version 26.0 software (IBM, Armonk, New York). The Medical Research Ethics Committee of Utrecht University Medical Center waived ethical approval for this study.

### Evaluating Different Hypotheses Using Simulation Models

We modified our previously published age-stratified RSV transmission model that accounts for population dynamics, RSV seasonality, and virus importation from external sources to account for waning immunity (Supplementary Text 1, Supplementary Figure 3, and Supplementary Table 2) [6]. Our previous model assumed that individuals gain partial immunity following infection, which reduces their susceptibility to subsequent infections. By adding waning immunity, we assumed that individuals can become susceptible to infection after a long period of low virus exposure.

Several factors could explain differences in the timing and intensity of RSV epidemics before the COVID-19 pandemic and after relaxation of COVID-19 restrictions, including length and strength of NPIs, RSV importation from external sources, increased birth rate in the Netherlands during COVID-19, and waning population immunity against RSV due to absent RSV circulation. We simulated RSV transmission from July 2018 to June 2025 to evaluate the various factors’ impact on the projected trajectories of RSV hospitalizations and qualitatively compared these to the observed reemergent RSV epidemic in the Netherlands. The 4 scenarios that we evaluated were (1) moderate level of NPIs and low level of virus importation, with no waning immunity; (2) moderate level of NPIs and low level of virus importation, with waning immunity; (3) moderate level of NPIs and high level of virus importation, with waning immunity; and (4) strict NPIs but high level of virus importation, with waning immunity (Supplementary Table 3). We developed a free-to-use web-based Shiny app to allow researchers to simulate reemergent RSV under the impact of various factors. We provide an interactive example at https://3wxpl3-zhe-zheng.shinyapps.io/shiny/. Data and code to reproduce this study are available from GitHub (https://github.com/weinbergerlab/SPREAD.git).

## RESULTS

### RSV-Related Pediatric Hospitalizations

**Figure 1.**
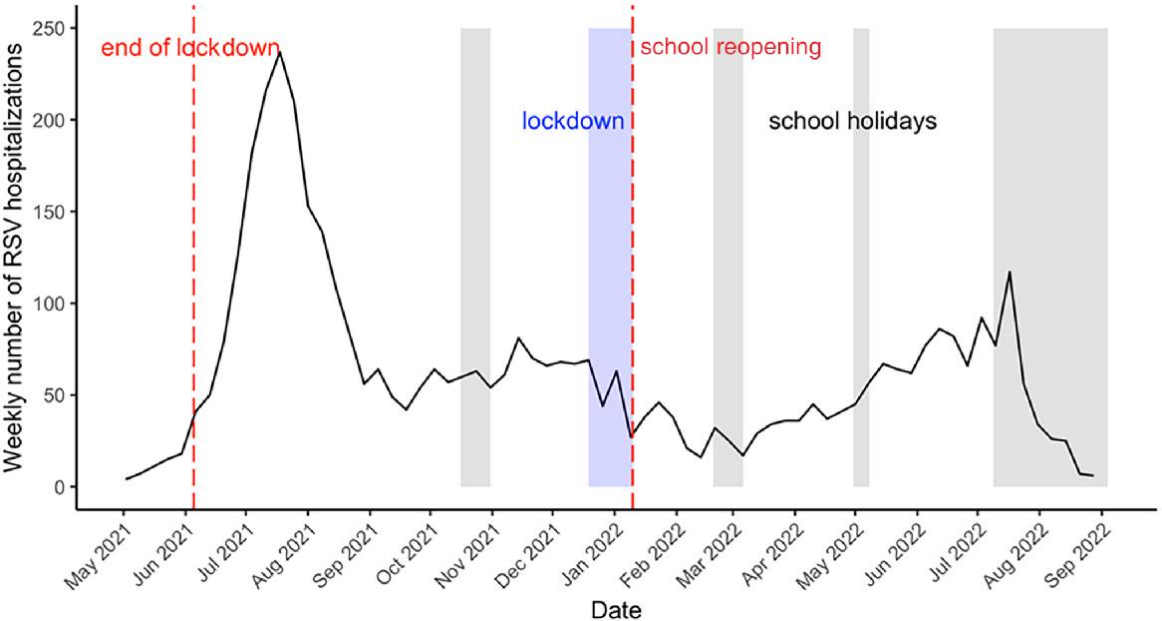
Weekly number of children aged <2 years admitted with respiratory syncytial virus (RSV) infection in the Netherlands between May 2021 and August 2022. Gray-shaded areas indicate period of school closures.

Starting from 24 May 2021 (week 21), a summer outbreak of RSV was observed. During the peak week (19 July 2021, week 29), 240 patients were admitted with RSV bronchiolitis. Subsequently, continuous RSV transmission was observed, with RSV-related admissions stabilizing at approximately 50 patients weekly (Figure 1).

### Older Patients During and After the 2021 Summer Outbreak

We obtained age-stratified data for 1269 patients aged <2 years admitted with RSV infection for 2018–2022 in 10 hospitals (Supplementary Figure 4). The proportion of patients aged <6 months was higher during the pre–COVID-19 winter seasons. The median age during pre–COVID-19 winter seasons 2018–2019 and 2019–2020 was 69 days (interquartile range [IQR], 34–175 days). During the summer outbreak, the median age increased to 161 days (IQR, 55–364 days) (*P* < .001). During the endemic phase (September 2021–August 2022), median age decreased again to 132 days (IQR, 54–253 days) but was still higher than during pre–COVID-19 winter seasons (*P* < .001; Supplementary Table 4). As a sensitivity analysis, we excluded patients from the only academic hospital (University Medical Center Utrecht), which contributed a large number of patients (20% pre–COVID-19 vs 8% during the summer outbreak and 8.5% during the endemic phase). Differences between groups remained unchanged (Supplementary Table 5).

### Association of Waning Population Immunity With RSV Epidemic Timing

**Figure 2.**
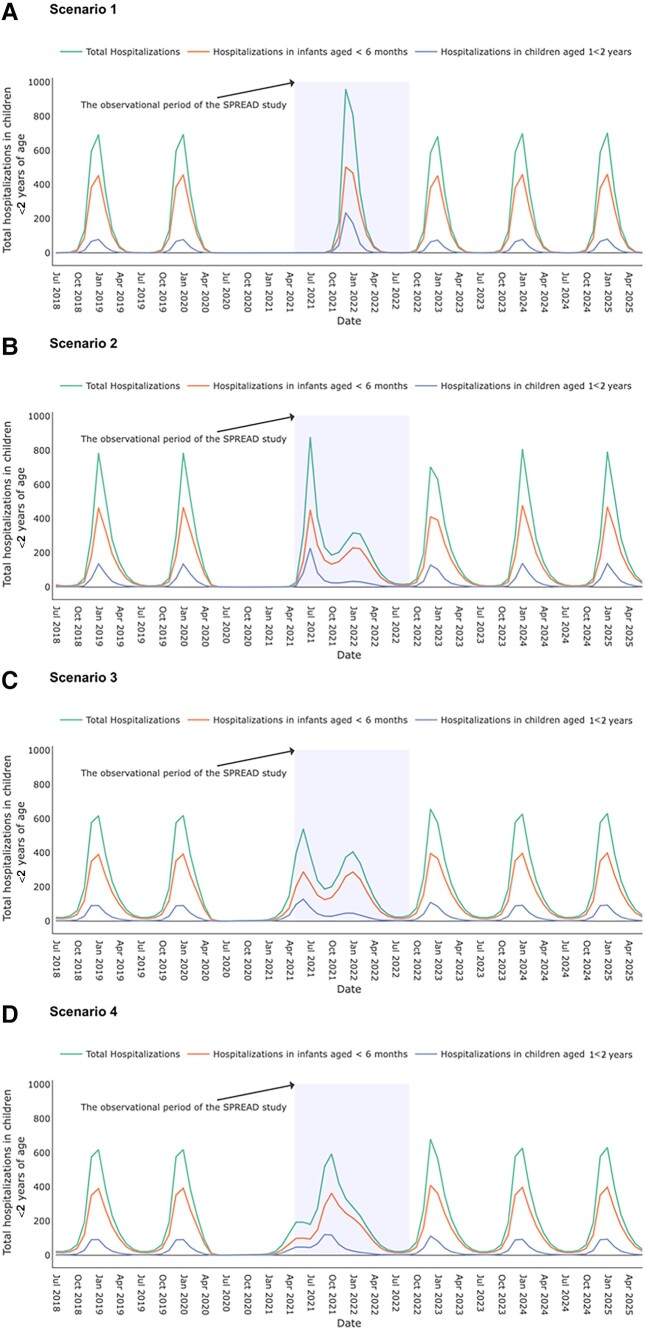
The simulated number of monthly respiratory syncytial virus (RSV) hospitalizations in children <2 years of age under 4 scenarios, 2018–2025. The projected RSV hospitalizations under 4 scenarios after the interruption of coronavirus disease 2019 (COVID-19)–related measures over time are plotted for July 2018 to June 2025. Scenario 2 corresponds to the simulated number of monthly RSV hospitalizations in children <2 years of age in the Netherlands. The green line corresponds to the total RSV hospitalizations in children <2 years of age; the orange line corresponds to RSV hospitalizations in infants <6 months of age; the purple line corresponds to RSV hospitalizations in children 1–<2 years of age. The shaded area corresponds to the period of the Surveillance of Pediatric Respiratory Admissions in Dutch Hospitals (SPREAD) study, May 2021–August 2022. *A*, Scenario 1: moderate level of nonpharmaceutical interventions (NPIs) and low level of virus importation, with no waning immunity. *B*, Scenario 2: moderate level of NPIs and low level of virus importation, with waning immunity. *C*, Scenario 3: moderate level of NPIs and high level of virus importation, with waning immunity. In previous scenarios, we assumed that COVID-19–related public health measures started in April 2020 and gradually relaxed since March 2021 over a 3-month period [7]. *D*, Scenario 4: strict NPIs but high level of virus importation, with waning immunity. Scenario 4 is similar to the observation in France and Germany with a few parameter adjustments. We assumed that COVID-19–related public health measures started at the end of March 2020 and gradually relaxed since the end of June 2021 over a 3-month period and that virus importation is 30 per 1 million travelers per month [7].

A model that assumed moderate NPIs, low virus importation, and waning population immunity against RSV due to low RSV circulation (scenario 2) most closely resembled the Dutch situation: a large summer outbreak followed by continuous RSV transmission (Figure 2*B*). Under this scenario, the proportion of RSV hospitalizations in children aged 1–<2 years was expected to be higher during the summer outbreak than during a typical winter season. This proportion decreased over time during the following “endemic” phase. Alternative scenarios that assumed no waning RSV immunity failed to generate a summer outbreak in 2021 that was more intense than previous winter epidemics (Figure 2*A*). A model that assumed strict NPIs, high level of virus importation, and waning immunity after a prolonged period of low viral exposure (scenario 4) most closely resembled RSV activity in Germany and France: RSV activity returned to that of normal winter epidemics after a small outbreak in spring 2021 (Figure 2*D*).

## DISCUSSION

The Netherlands exhibited a unique pattern of RSV reemergence during the COVID-19 pandemic characterized by a high summer peak in 2021 followed by a prolonged period of continuous transmission at mid- to high-level RSV activity. Our model simulations confirm that population “immunity debt” can explain the large RSV summer outbreak and the following “endemic phase” (Supplementary Figure 5) [4, 8–10]. Additionally, NPIs were gradually reimplemented between mid-November 2021 and the end of January 2022. This, combined with school holidays in December and mid-February, may also have contributed to a stagnation of RSV activity.

The year-round continuous RSV transmission pattern is distinctive not only from prepandemic winter epidemics in the Netherlands but also from neighboring countries such as Germany and France. These 2 countries returned to a winter epidemic in 2021 with low RSV activity during the summer of 2022. NPIs strictness and virus importation from external sources could explain these observed variations in RSV seasonality as these factors shape the level of population immunity debt [6, 10]. Furthermore, differences in surveillance and reporting strategies may explain the observed difference.

Our results suggest that although a shift in RSV seasonality may occur after implementation and subsequent relaxation of NPIs, RSV activity will most likely return to normal epidemic timing because herd immunity against RSV infection will return to prepandemic levels after 2 seasons of RSV exposure. To date, our model projection is aligned with the national RSV activity report from the Dutch National Institute for Public Health and the Environment [11].

Our study has several limitations. First, since not all participating hospitals used standard RSV testing, we may have underestimated RSV-related hospitalization rates. Some hospitals only used standard testing during the summer outbreak, which may have led to the underestimation of hospitalizations after the outbreak. Second, data collection was not standardized for each hospital. Although most hospitals shared prospective data collected at the clinical ward, some used RSV-specific DBC codes or virology results that were manually checked to match the inclusion criteria. Third, we received consistent data from 34 of 46 hospitals for the entire study period; this may have led to underestimation of hospitalization rates. Fourth, our model was not calibrated to the observed hospitalization data in the Netherlands due to the short observational period. Instead, we simulated RSV epidemics using parameters from previous RSV models in the United States based on Dutch demographics. Although our simulations may not perfectly resemble the reemergent RSV epidemics in the Netherlands, the flexible parameter ranges in our web-based app provide an opportunity for researchers from other countries to simulate reemergent epidemics based on their local demographic and epidemiological characteristics. Finally, as no real-time RSV hospital surveillance system existed before the start of this study, we were not able to compare our data to data from previous seasons.

In summary, we describe a distinct pattern of ongoing RSV transmission following an out-of-season RSV outbreak in the Netherlands after relaxation of COVID-19–related NPIs. The pattern is different from neighboring countries and may be partly explained by “immunity debt”—that is, waning population immunity after a long period of low RSV exposure. Continuous monitoring of RSV seasonality using hospital-based data is essential to anticipate future RSV epidemics.

## Supplementary Data

Supplementary materials are available at *The Journal of Infectious Diseases* online. Consisting of data provided by the authors to benefit the reader, the posted materials are not copyedited and are the sole responsibility of the authors, so questions or comments should be addressed to the corresponding author.

## Supplementary Material

jiad282_Supplementary_Data

## Notes

***Acknowledgments.*** The authors thank the children and their families who participated in the study; Eline Bel, Katja Steenhuis, Marit de Bruijne, Kiora Russel, Nathalie Oldenburger, Kayleigh Vievermanns, Kim Bodaar, and Arda Yilmaz for their assistance in data collection; and all nurses, nurse practitioners, supporting staff, doctors, and pediatricians for their substantial and ongoing effort for this study. Last, the authors thank the Dutch Working Group on Clinical Virology from the Dutch Society for Clinical Microbiology and all participating laboratories for providing the virological data from the weekly laboratory virological report.

***SPREAD Study Group Authors.*** Marlies Vermaas-van Putten (Admiraal de Ruyter Ziekenhuis); Elly Smit-Kleinlugtenbeld (Albert Schweitzer Ziekenhuis); Marieke Peetsold (Alrijne Ziekenhuis); Martijn van der Kuip, Hans van Goudoever, and Britt van Keulen (Amsterdam Universitair Medisch Centrum); Anouk Boot and Robin Kloos (St Antonius Ziekenhuis); Sandy van Gool (Antonius Zorggroep Sneek); Yvonne Snepvangers (Beatrix Ziekenhuis); Anke Kuijpers (Bernhoven Ziekenhuis); Negassi Menelik (BovenIJ Ziekenhuis); Stephanie de Crom (Bravis Ziekenhuis); Carien Miedema (Catharina Ziekenhuis); Gavin ten Tusscher and Jet van Giessen (Dijklander Ziekenhuis); Ronald de Moor (Elizabeth-TweeSteden Ziekenhuis); Marianne Faber and Mijke Breukels (Elkerliek Ziekenhuis); Vincent Jaddoe and Liesbeth Duijts (Erasmus Medisch Centrum); Claire Lutterman (Flevoziekenhuis); Ilka Vink and Gerdien Tramper-Stranders (Franciscus Gasthuis & Vlietland); Annemarie Oudshoorn and Astrid Ritman (Gelre Ziekenhuis); Gerdien Dubbink-Verheij (Groene Hart Ziekenhuis); Jantien Bolt and Cagri Cakir (Haaglanden Medisch Centrum); Edwin Rietveld (IJsselland Ziekenhuis); Jolita Bekhof (Isala); Edmond Rings and Jara de Swart (Leids Universitair Medisch Centrum); Gertjan Driessen (Maastricht University Medical Center); Rienus Doedens (Martini Ziekenhuis); Lieke Nijssen and Lonneke van Onzenoort-Bokken (Maxima Medisch Centrum); Ruud Meijneke (Meander Medisch Centrum); Machteld van Scherpenzeel and Tina Faber (Medisch Centrum Leeuwarden); Femke de Groof and Sarah Schouten (Noordwest Ziekenhuisgroep); Julia van de Zande and Monique op de Coul (Onze Lieve Vrouwe Gasthuis); Stefanie Henriet and Kim Stol (Radboud Universitair Medisch Centrum); Maaike van Rossem (Rijnstate Ziekenhuis); Monique Jacobs (Slingeland Ziekenhuis); Marlies van Houten (Spaarne Gasthuis); Roy Zuurbier and Frans Plötz (Tergooi Medisch Centrum); Andra de Vries (Treant Zorggroep); Rinske van der Heide (Universitair Medisch Centrum Groningen); Anneke van Boekholt (VieCuri); Gieneke Gonera de Jong (Wilhelmina Ziekenhuis Assen); Amara Nassar-Sheikh Rashid and Manouck Roelofs (Zaans Medisch Centrum); Károly Illy (Ziekenhuis Rivierenland); Naomi Reijmerink (Ziekenhuis St Jansdal); Stefan van Dorth (Ziekenhuis Tjongerschans); Saskia Schipper (ZorgSaam); Philippe Rosias (Zuyderland Medisch Centrum); and Anne Teirlinck (Rijksinstituut voor Volksgezondheid en Milieu).

***Financial support.*** This work was supported by ZonMw (The Netherlands Organization for Health Research and Development) (50-55700-98-001) and the National Institute of Allergy and Infectious Diseases (MIDAS Program) of the NIH (award number R01AI137093).
